# Are Cardiovascular Risk Factors also Associated with the Incidence of Atrial Fibrillation? A Systematic Review and Field Synopsis of 23 Factors in 32 Population-Based Cohorts of 20 Million Participants

**DOI:** 10.1160/TH16-11-0825

**Published:** 2017-02-23

**Authors:** Victoria Allan, Shohreh Honarbakhsh, Juan-Pablo Casas, Joshua Wallace, Ross Hunter, Richard Schilling, Pablo Perel, Katherine Morley, Amitava Banerjee, Harry Hemingway

**Affiliations:** 1 Farr Institute of Health Informatics Research, Institute of Health Informatics, University College London, London, UK; 2 The Barts Heart Centre, St Bartholomew’s Hospital, Barts Health NHS Trust & Queen Mary, University of London, London, UK; 3 Centre for Global Non Communicable Diseases, London School of Hygiene & Tropical Medicine, London, UK; 4 Institute of Psychiatry, Psychology & Neuroscience, Kings College London, London, UK

**Keywords:** Atrial fibrillation, risk factors, primary prevention, clinical guidelines, cardiovascular disease

## Abstract

Established primary prevention strategies of cardiovascular diseases are based on understanding of risk factors, but whether the same risk factors are associated with atrial fibrillation (AF) remains unclear. We conducted a systematic review and field synopsis of the associations of 23 cardiovascular risk factors and incident AF, which included 84 reports based on 28 consented and four electronic health record cohorts of 20,420,175 participants and 576,602 AF events. We identified 3-19 reports per risk factor and heterogeneity in AF definition, quality of reporting, and adjustment. We extracted relative risks (RR) and 95 % confidence intervals [CI] and visualised the number of reports with inverse (RR [CI]<1.00), or direct (RR [CI]>1.00) associations. For hypertension (13/17 reports) and obesity (19/19 reports), there were direct associations with incident AF, as there are for coronary heart disease (CHD). There were inverse associations for non-White ethnicity (5/5 reports, with RR from 0.35 to 0.84 [0.82–0.85]), total cholesterol (4/13 reports from 0.76 [0.59–0.98] to 0.94 [0.90–0.97]; 8/13 reports with non-significant inverse associations), and diastolic blood pressure (2/11 reports from 0.87 [0.78–0.96] to 0.92 [0.85–0.99]; 5/11 reports with non-significant inverse associations), and direct associations for taller height (7/10 reports from 1.03 [1.02–1.05] to 1.92 [1.38–2.67]), which are in the opposite direction of known associations with CHD. A systematic evaluation of the available evidence suggests similarities as well as important differences in the risk factors for incidence of AF as compared with other cardiovascular diseases, which has implications for the primary prevention strategies for atrial fibrillation.

## Introduction

Atrial fibrillation (AF) is the world’s most common heart rhythm disorder, affecting 33.5 million people globally in 2010 (1). AF accounts for 1 in 4 ischaemic strokes (2), doubles the risk of death (3), places an economic burden on healthcare systems (4), and is projected to affect twice as many people by 2050 (5, 6). Yet to date, there have been no clinical trials of healthy participants without cardiovascular disease (CVD), and with AF as the primary outcome (7). The focus of trials has instead been on prevention of stroke and thromboembolism after diagnosis of AF. Community screening programmes for detection of AF (8), are also designed to identify patients at high risk of stroke and thromboembolism, and do not identify those who are at an initially high risk of later developing AF. Thus, current clinical guidelines make no recommendations for the primary prevention of AF itself, among people without CVDs (9–11).

Established primary prevention strategies of other CVDs, such as coronary heart disease (CHD) (12), and stroke (13), are based on understanding of risk factors, but the extent to which the same risk factors are associated with the incidence of AF is not fully understood. Ultimately, it is not known whether existing CVD prevention strategies can also work in preventing AF, or whether there may be important clinical differences. In synthesising available evidence the conventional (near universal) approach is to examine risk factors one at a time. Single risk factor systematic reviews and meta-analyses have been carried out for alcohol (14–16), C–reactive protein (17), diabetes mellitus (18), obesity (19), physical activity (20, 21), and renal function (22) in relation to AF risk. Each of these reviews uses non-identical methods, for example varying in the extent to which incident AF is analysed among people free from pre-existing CVD. While there is an important ongoing role for the vertical approach of a single risk factor meta-analysis (particularly if methods can be aligned), there is also a complementary role for a horizontal ‘field synopsis’ approach across multiple potential risk factors. The term field synopsis is defined as a systematic evaluation of evidence in which the i) overall amount, ii) extent of replication, and iii) protection from bias is considered across the whole field (23, 24). One advantage of a field synopsis in multifactorial diseases is to provide an unbiased empirical basis for prioritising further research into risk factors with preventive potential.

We therefore conducted a systematic review and field synopsis of the associations of a wide range of demographic, behavioural, and biological CVD risk factors and incidence of AF in general populations and populations initially free from diagnosed CVD. Field synopses’ of cumulative evidence (23, 24), are common in genetics but have seldom been applied in the context of preventive medicine. Our objectives were i) to determine the amount of evidence for each risk factor, ii) to evaluate the extent to which each risk factor shows concordant or discordant associations with AF incidence across independent study populations, and iii) to systematically appraise the quality of the observational evidence across the field of AF prevention research.

## Methods

Our approach to the search, selection, data collection and analysis of reports was systematic, and guided by the Preferred Reporting Items for Systematic reviews and Meta-Analyses (PRISMA) checklist (25).

### Search strategy

We queried the PubMed database using the search terms listed in the Appendix p 3, for original research reports that were published in English up to October 1, 2015; involving prospective, population based cohorts that were either initially free from diagnosed CVD at baseline or were general population cohorts in which the proportion of people with diagnosed CVD at baseline was low reflecting prevalence in the general population. Cohorts were of any age, and without prior AF; and investigated the association between “risk factors” and incident AF, over any follow–up period, and using Cox proportional hazards or Poisson regression models adjusted or stratified for age and sex as a minimum. We shortlisted 23 cardiovascular risk factors (listed in ► Table 1) for review, based on clinical relevance as an established predictor or treatment target in the prevention of CVD (12), or on clinical opinion of an association with AF (9), and on expert consensus between authors. Reference lists of identified reports, existing reviews and meta–analyses (which were not restricted to prospective cohorts of individuals either free from or with general population levels of baseline CVD: alcohol (14–16), C–reactive protein (17), diabetes mellitus (18), obesity (19), physical activity (20, 21), and renal function (22)), were hand–searched for additional reports. Two out of three authors (JW, SH, VA) reviewed the inclusion of each report based on title, then abstract, then full–text. Disagreements were resolved by joint full–text review with a third independent reviewer (RH).

### Data extraction

From each report the following information was extracted: design of cohort (consented participant cohort with research measures at baseline and follow-up, or electronic health record (EHR) cohort in which anonymised data collected as part of usual clinical care was used for baseline and follow–up measures), country, sample size (number of participants at baseline) and number of AF events over follow–up (based on the highest figure reported), age range, proportion of female participants, mean or median follow–up, methods of AF ascertainment, risk factor definition, statistical model, and risk factors used in adjustment. We extracted data on whether cardiovascular events, prevalent at baseline and incident during follow–up and preceding AF were accounted for. For each risk factor, we extracted adjusted relative risks (RR), and 95 % confidence intervals [95 % CI]. Where there were multiple RR reported within a publication, or across multiple publications from the same cohort, we selected the most adjusted estimate, modelled with the highest number of AF cases.

### Summary and visualisation of risk factor associations

We summarised the overall results of the field of cohort epidemiology of AF by plotting the number of reports with inverse (RR<1.00), null or mixed (RR=1.00 or shows opposite associations among subpopulations), or direct relationship (RR>1.00) with AF incidence. We regarded the association as significant if the 95 % CI did not cross unity. Unless stated, RR are given as originally reported. For each factor, we then plotted the RR and 95 % CI using R–3.2.0 (CALIBERdatamanage package, available at: caliberresearch.org).

### Summary and visualisation of quality of reporting and analysis

We summarised the quality of reporting by completeness of the items listed in the above data extraction section (items not reported (NR) are clearly indicated in tables and figures). We summarised the quality of analysis by assessment of the number (%) of adjustment made for the 23 risk factors, and whether adjustment was made for six standard CVD risk factors (age, sex, smoking, blood pressure, lipids and diabetes mellitus), and for prevalent and incident CVD events. We visualised these as “Swiss cheese” plots (26).

## Results

### Characteristics of included reports

Out of 2777 publications, 73 were included (Suppl. Figure S1, Appendix p 4) with 84 reports on 32 cohorts from 10 countries and 20,420,175 participants (16, 27–98). As ► Table 2 (available online at www.thrombosis-online.com) shows, 28 (87.5 %) cohorts involved consented participants with 39,900 (6.9 %) events, and four cohorts (12.5 %) were EHR–based with 536,702 (93.1 %) events. AF events were ascertained from a research electrocardiogram (40 reports (47.6 %)), diagnosis codes from medical records (60 reports (71.4 %)), or using a combination of both methods (24 reports (28.6 %)). As Suppl. Table S1 (Appendix pp 5–7) shows, 17 reports (20.2 %) described using two out of four types of medical records (i. e. general practitioner, hospital care, prescriptions, or mortality records), but no report used three or all four types combined.

### Quality of reporting

Age range was not reported in 30 reports (35.7 %), mean or median follow–up in 18 reports (21.4 %), and risk factor definition was not reported in nine reports (10.7 %). Information was consistently reported on country, sample size, female participants, and AF events.

### Quality of analysis

Overall, 63 reports (75.0 %) lacked adjustment for all six standard CVD risk factors (Suppl. Table S2, Appendix pp 8–9). Age was adjusted for in 84 reports (100.0 %), sex in 80 reports (95.2 %), smoking in 49 reports (58.3 %), blood pressure in 63 reports (75.0 %), lipids in 32 reports (38.1 %), and diabetes mellitus in 59 reports (70.2 %). The total number of adjustment factors ranged from 2–14 factors, with a median of eight factors. There was lack of adjustment for prevalent CVD in 30 reports (35.7 %), and for incident CVD in 69 reports (82.1 %).

### Associations of 23 risk factors and incidence of AF

A summary of the heterogeneity of associations of 23 risk factors and incidence of AF are visualised in ► Figure 1, and for each factor separately in ► Figures 2–6 and Suppl. Figures S2–S19 (Appendix pp 12–29). There was no evidence of small study bias.

### Demographic factors

**Figure 2:**
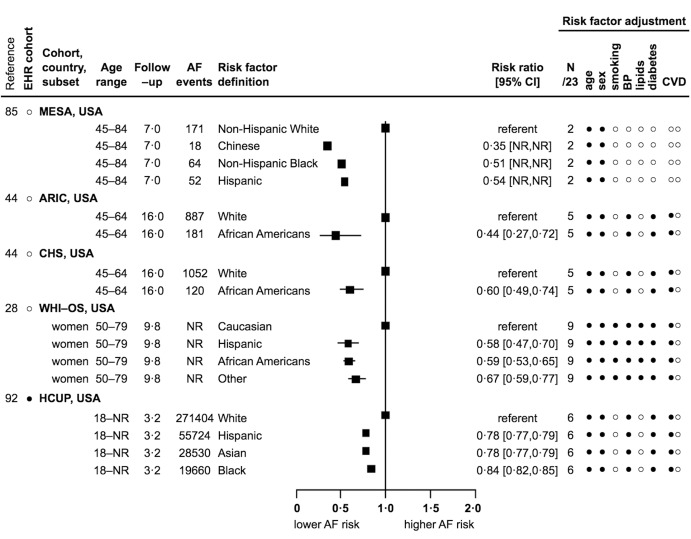
**Association of ethnicity and incidence of AF: 5 reports from 1 country with 386,115 events.** EHR – electronic health record, age range in years, follow-up in years (mean, median, or maximum), AF – atrial fibrillation, CI – confidence interval, N/23 – number (of factors) out of 23, CVD – cardiovascular disease, SD – standard deviation, NR – not reported, USA – United States of America, • – yes, ○ – no, -- – not applicable. Risk factor adjustment refers to whether adjustment was made for the 23 risk factors under review, 6 CVD risk factors, and prevalent and incident CVD events. Example: ARIC adjusted for 5/23 risk factors, age, sex, blood pressure (i. e. any of systolic blood pressure, diastolic blood pressure, hypertension, or blood pressure lowering medication), and diabetes mellitus, but not smoking or lipids (i. e. any of total cholesterol, low–density lipoprotein cholesterol, high–density lipoprotein cholesterol, triglycerides, hyperlipidaemia, or lipid lowering medication), and prevalent, but not incident CVD events. For cohort abbreviations see Table 2 (available online at www.thrombosis-online.com).

For age, all 15 reports showed significant direct associations, but these were heterogeneous. RR [95 %CI] ranged from 1.02 [1.01–1.03] to 1.14 [1.10–1.18] for every 1–year, from 1.43 [1.29–1.59] to 1.65 [1.57–1.74] for every 5–year, from 1.09 [1.09–1.09] to 2.35 [2.03–2.72] for every 10–year, and from 1.36 [1.27–1.45] to 4.34 [3.72–5.07] for every standard deviation (NR) year increase in age (Suppl. Figure S2 in Appendix) (28, 32, 35, 37, 43, 47, 50, 55, 67, 70, 88, 90, 94, 98). For men (compared to women), one report showed a significant inverse association (0.70 [0.50–0.90]) (79), two reports were inverse but non-significant (from 0.95 to 0.96) (88, 98), and eight reports showed significant direct associations (from 1.45 [1.29–1.63] to 1.90 [1.58–2.29]) (Suppl. Figure S3 in Appendix) (37, 43, 47, 50, 55, 70, 94). For African-American, Asian, Chinese, Hispanic and Non-Hispanic Black (compared to White) ethnicities, all five reports showed significant inverse associations (from 0.35 [NR–NR] to 0.84 [0.82–0.85]) (28, 44, 85, 92). Only one country reported estimates for the association of ethnicity and incidence of AF (► Figure 2). For socio–economic status, two reports showed significant inverse associations, three reports were inverse but non-significant, and one report showed a mixed association (see Appendix p 10 and Suppl. Figure S4 for further details).

### Health behaviours

**Figure 3:**
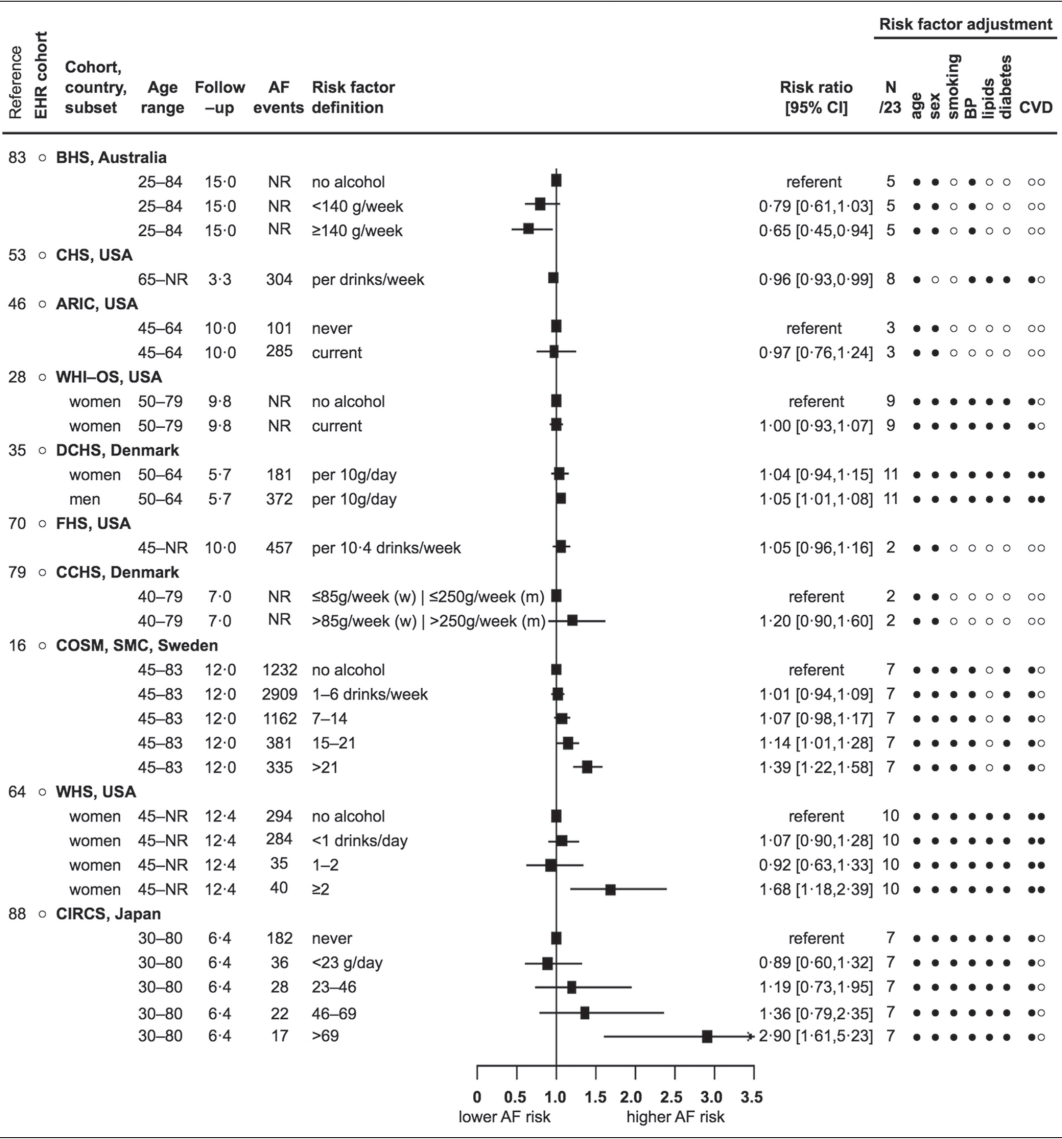
**Association of alcohol intake and incidence of AF: 10 reports from 5 countries with 18,997 events.** Legend see Figure 2 abbreviations, and g – grams, (w) – women, (m) – men.

For current smoking, one report was inverse but non-significant (0.78) (35), one report showed a mixed association (47), five reports were direct but non-significant (from 1.01 to 1.20) (54, 56, 70, 83, 88), and six reports showed significant direct associations (from 1.32 [1.19–1.46] to 2.00 [1.40–2.80]) (Suppl. Figure S5 in Appendix, available online at www.thrombosis-online.com) (28, 37, 40, 47, 78, 79). For physical activity, three reports showed significant inverse associations, four reports were inverse but non-significant, two reports showed null or mixed associations, and two reports were direct but non-significant (see Appendix p 10 and Suppl. Figure S6). For alcohol intake in drinks per day or week, in grams per day or week, or for current alcohol drinkers, two reports showed significant inverse associations (from 0.65 [0.45–0.94] to 0.96 [0.93–0.99]) (53, 83), one report was inverse but non-significant (0.97) (46), one report showed a null association (28), three reports were direct but non-significant (from 1.04 to 1.20) (35, 70, 79), and three reports showed significant direct associations (from 1.39 [1.22–1.58] to 2.90 [1.61–5.23]) (16, 64, 88). All 10 alcohol reports defined alcohol intake differently, and as shown for the three direct alcohol associations, the increased risk of developing AF was only among the highest alcohol intake categories (► Figure 3).

### Blood pressure

**Figure 4:**
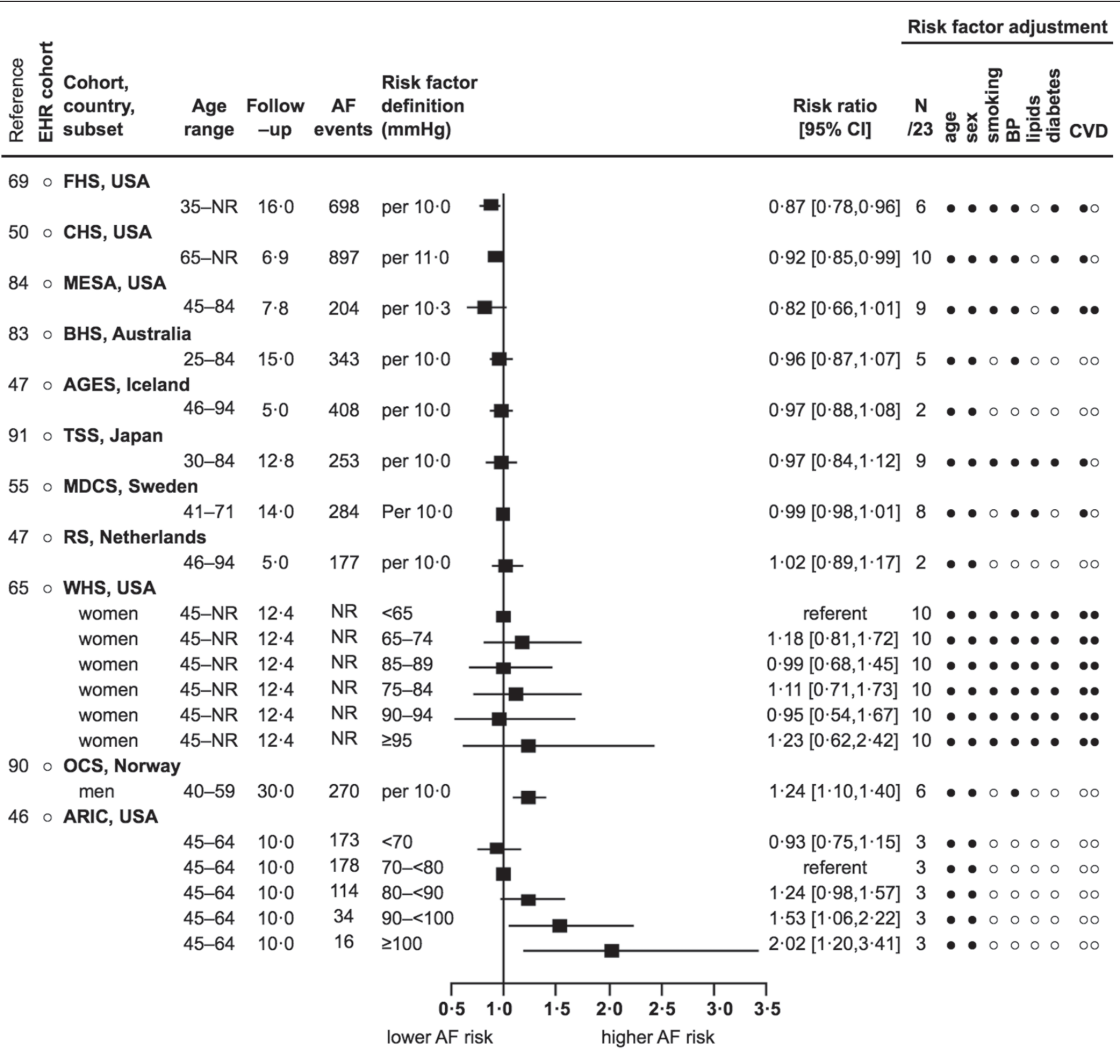
**Association of diastolic blood pressure and incidence of AF: 11 reports from 7 countries with 4796 events.** See Figure 2 abbreviations, and mmHg – millimetres of mercury. Risk factor adjustment for BP in this instance refers to whether systolic blood pressure, hypertension, or blood pressure lowering medication were adjusted for.

For every 10–22 mmHg increase in systolic blood pressure, or systolic blood pressure ≥160 mmHg, one report showed a null association (79), five reports were direct but non-significant (from 1.01 to 1.24) (35, 47, 55, 83, 84), and eight reports showed significant direct associations (from 1.14 [1.05–1.25] to 2.63 [1.83–3.78]) (Suppl. Figure S7 in Appendix) (46, 47, 50, 56, 65, 69, 90, 91). For every 10–11 mmHg increase in diastolic blood pressure, or diastolic blood pressure ≥95–100 mmHg, two reports showed signifitein cant inverse associations (from 0.87 [0.78–0.96] to 0.92 [0.85–0.99]) (50, 69), five reports were inverse but non-significant (from 0.82 to 0.99) (47, 55, 83, 84, 91), two reports were direct but non-significant (from 1.02 to 1.23) (47, 65), and two reports showed significant direct associations (from 1.24 [1.10–1.40] to 2.02 [1.20–3.41]) (46, 90). No EHR cohorts reported estimates for the association of diastolic blood pressure and incidence of AF (► Figure 4). For hypertension, one report was inverse but non-significant (0.93) (88), three reports were direct but non-significant (from 1.21 to 1.37) (35, 55, 79), and 13 reports showed significant direct associations (from 1.28 [1.08–1.51] to 2.60 [1.60–4.40]) (Suppl. Figure S8 in Appendix) (28, 31, 37, 40, 47, 50, 56, 67, 70, 87, 91, 98).

### Lipid profile

**Figure 5:**
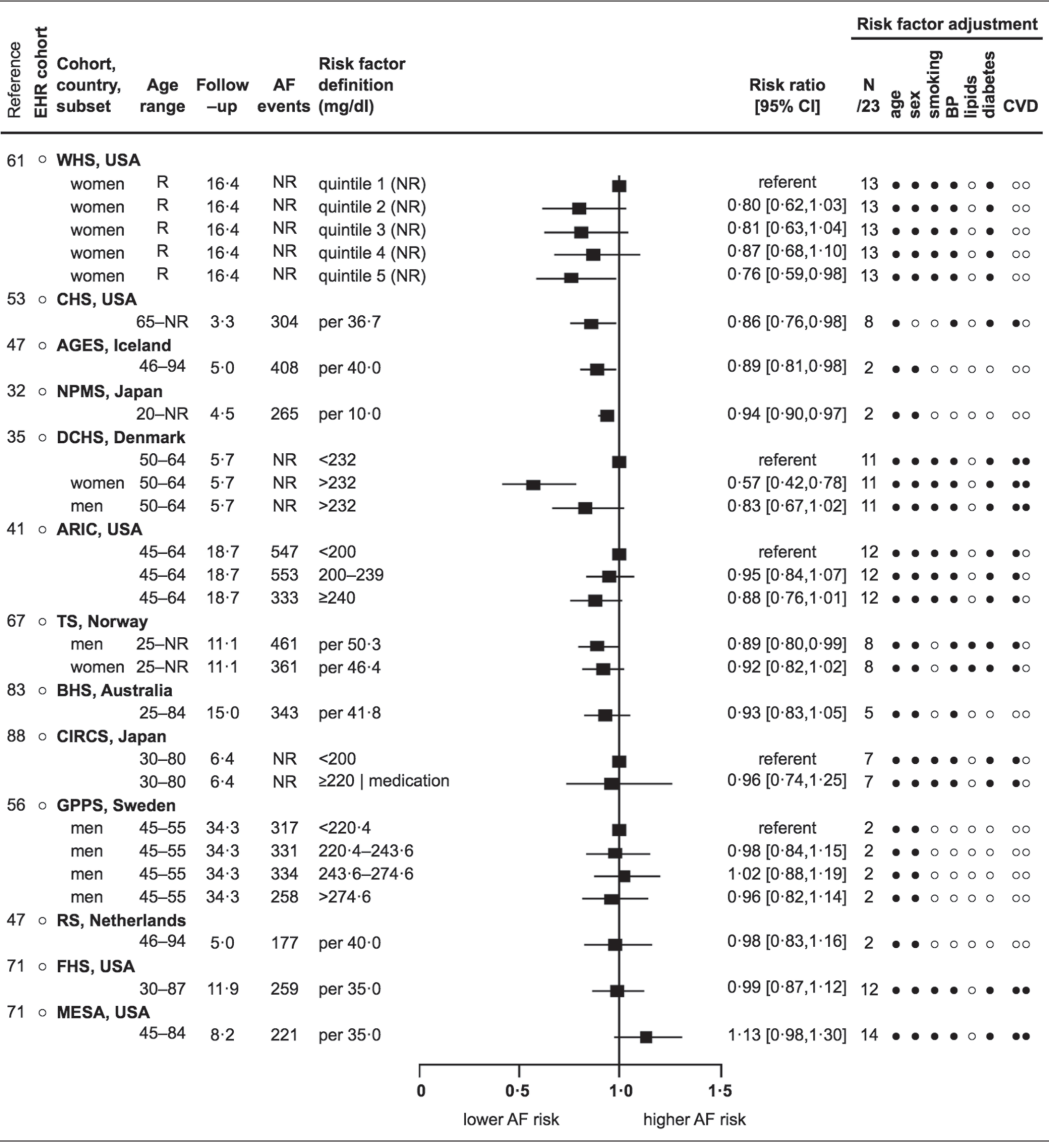
**Association of total cholesterol and incidence of AF: 13 reports from 8 countries with 7129 events.** See Figure 2 abbreviations, and mg/dl – milligrams per decilitre, mmol/l – millimoles per litre. Risk factor adjustment for lipids in this instance refers to whether low–density lipoprocholesterol, high–density lipoprotein cholesterol, triglycerides, hyperlipidaemia, or lipid lowering medication were adjusted for. Total cholesterol reported as mmol/l for CHS, GPPS, TS and BHS was converted to mg/dl using the conversion 1 mmol/l = 38.66976 mg/dl.

For every 10–50 mg/dl increase in total cholesterol, or total cholesterol ≥220–280 mg/dl, four reports showed significant inverse associations (from 0.76 [0.59–0.98] to 0.94 [0.90–0.97]) (32, 47, 53, 61), eight reports were inverse but non-significant (from 0.57 to 0.99) (35, 41, 47, 56, 67, 71, 83, 88), and one report was direct but non-significant (1.13) (71). Both inverse and direct associations were shown in the three total cholesterol reports that adjusted for prevalent and incident CVD events (► Figure 5). For every 10–40 mg/dl increase in low–density lipoprotein cholesterol, or low–density lipoprotein cholesterol ≥150 mg/dl, two reports showed significant inverse associations (from 0.72 [0.56–0.92] to 0.92 [0.88–0.96]) (32, 61), four reports were inverse but non-significant (from 0.85 to 0.95) (41, 55, 71, 83), and one report was direct but non-significant (1.15) (Suppl. Figure S9 in Appendix) (71). For every 15 mg/dl increase in high–density lipoprotein cholesterol, or high–density lipoprotein cholesterol ≥60mg/dl, five reports were inverse but non-significant (from 0.85 to 0.98) (32, 47, 71), two reports showed null or mixed associations (41, 47), two reports were direct but non-significant (from 1.01 to 1.07) (61, 83), and one report showed a significant direct association (1.16 [1.04–1.28]) (Suppl. Figure 10 in Appendix) (67). For triglycerides, three reports were inverse but non-significant, one report showed a mixed association, two reports were direct but non-significant, and three reports showed significant direct associations (see Appendix p 10 and Suppl. Figure S11).

### Diabetes mellitus, renal function

For diabetes mellitus (type unspecified), two reports were inverse but non-significant (from 0.86 to 0.98) (83, 98), eight reports were direct but non-significant (from 1.02 to 1.49) (37, 47, 54, 56, 58, 67, 70), and six reports showed significant direct associations (from 1.17 [1.16–1.19] to 1.80 [1.30–2.60]) (Suppl. Figure S12 in Appendix) (28, 40, 50, 79, 88, 95). For renal function, three reports were inverse but non-significant, five reports were direct but non-significant, and three reports showed significant direct associations (see Appendix p 11 and Suppl. Figure S13).

### Anthropometric factors

**Figure 6:**
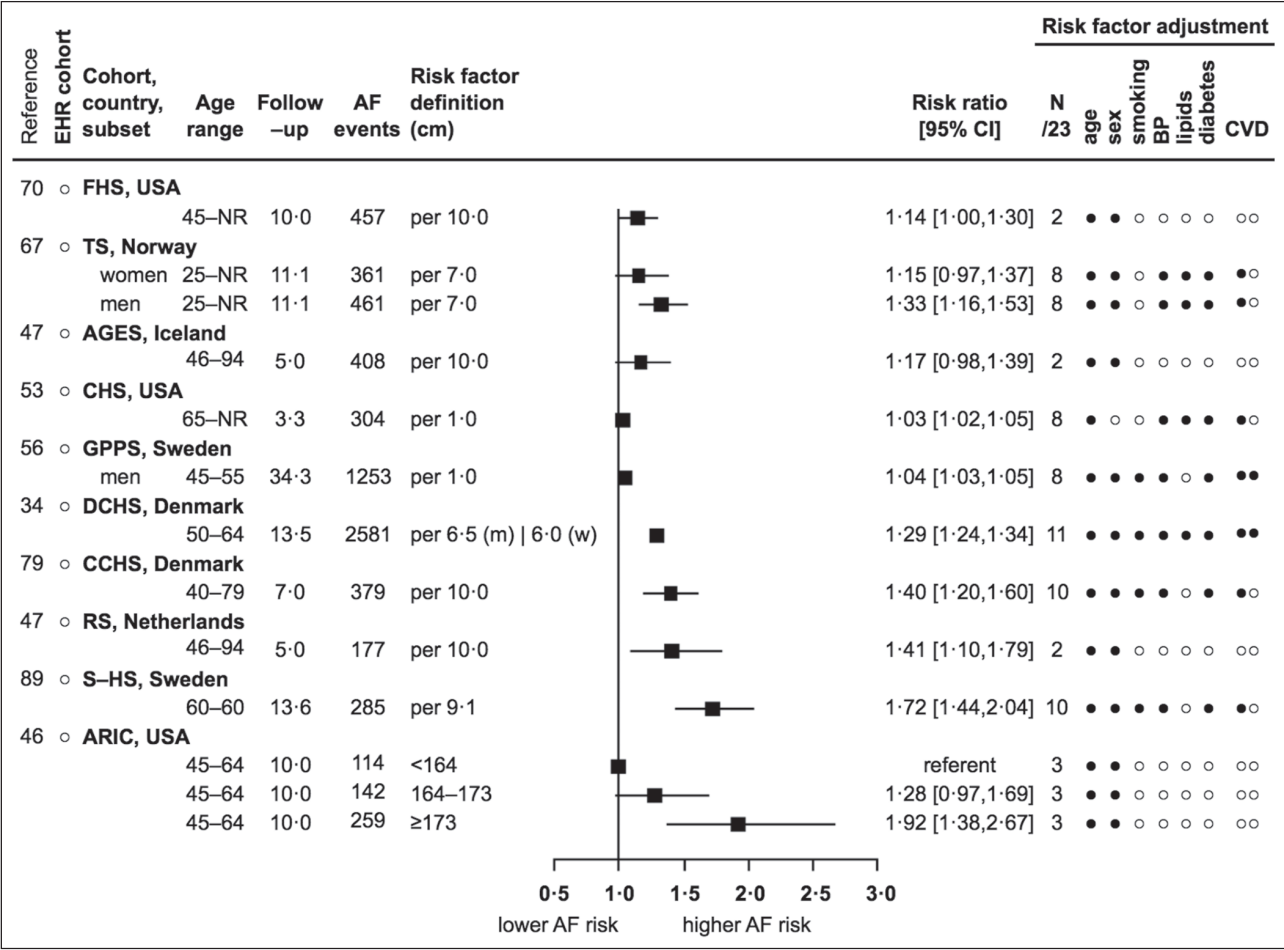
**Association of height and incidence of AF: 10 reports from 6 countries with 7181 events.** See Figure 2 abbreviations, and cm – centimetres, (m) – men, (w) – women.

For every 1–10 cm increase in height, or height ≥173 cm, three reports were direct but non-significant (from 1.14 to 1.17) (47, 67, 70), and seven reports showed significant direct associations (from 1.03 [1.02–1.05] to 1.92 [1.38–2.67]) (34, 46, 47, 53, 56, 79, 89) (► Figure 6). For weight, all eight reports showed significant direct associations (see Appendix p 11 and Suppl. Figure S14). For every 1–10 kg/m^2^ increase in body mass index (BMI), or BMI ≥25–30 kg/m^2^, all 19 reports showed significant direct associations (from 1.04 [1.02–1.05] to 2.24 [1.41–3.58]) (Suppl. Figure S15 in Appendix) (28, 31, 34, 37, 39, 48, 55, 56, 60, 67, 70, 76, 79, 81, 83, 88–91).

### Inflammatory biomarkers

For C–reactive protein, four reports were direct but non-significant, and four reports showed significant direct associations (see Appendix p 11 and Suppl. Figure S16). For fibrinogen, two reports were inverse but non-significant, one report was direct but non-significant, and three reports showed significant direct associations (see Appendix p 11 and Suppl. Figure S17).

### Thyroid function, autoimmune disease

For every 1.0 mU/l decrease in thyroid stimulating hormone, or thyroid stimulating hormone <0.10–0.45 mU/l, one report was inverse but non-significant (0.34) (82), five reports were direct but non-significant (from 1.06 to 2.85) (51, 77, 82), and two reports showed significant direct associations (from 1.41 [1.25–1.59] to 3.10 [1.70–5.50]) (Suppl. Figure S18 in Appendix) (72, 96). For autoimmune diseases, all three reports showed significant direct associations (see Appendix p 11 and Suppl. Figure S19).

### Discussion

To our knowledge this is the first example of a field synopsis evaluating associations across multiple risk factors and disease incidence. We systematically evaluated 84 reports from 32 independent cohorts for the impact of 23 cardiovascular risk factors on incidence of AF. Unlike previous overviews of AF risk factors (10, 99), we focussed exclusively on primary prevention among populations initially free from diagnosed CVD or general populations in which baseline levels of CVD reflected prevalence in the general population. We found some evidence that ethnicity, height, diastolic blood pressure and serum cholesterol, are associated with AF incidence in opposite directions to their known associations with CHD and stroke. Furthermore we found only modest evidence for the widely held clinical opinion that excess alcohol is associated with risk of AF. Taken together our findings suggest that primary prevention strategies for AF may require some different elements from the current approaches used for other CVDs.

### Concordant associations

For some risk factors – hypertension, and higher BMI – there were consistent, direct associations with incident AF, as there are for CHD. This could reflect a causal link with AF, or that the risk factor causes CHD, which in turn causes AF. Surprisingly, we found that only three (out of 14) reports investigating the association between systolic blood pressure and incident AF accounted for both prevalent and intercurrent incident cardiovascular events, and only one of which reported a significant direct association. Several post-hoc analyses of trials have suggested a possible benefit of ACE/ARB–inhibitors (100), and other blood–pressure lowering medications (101), for prevention of AF. However, we demonstrate that across all 23 risk factors, the available observational evidence does not fully consider a mechanism or confounding of reported associations by intercurrent CHD.

Current clinical guidelines include alcohol in a list of potentially “reversible” causes of AF, but acknowledge that there is no evidence to suggest addressing any of these is effective in preventing AF (9). We found a small number of reports (3 out of 10) suggesting a direct association between alcohol intake and AF incidence. This is in contrast to three existing alcohol reviews (Samokhvalov et al. (14), Kodama et al. (15) and Larsson et al. (16)), which have reported dose–response relationships. There are several possible explanations as to why our findings differ. Unlike the previous alcohol reviews, ours considers i) only prospective studies (Samokhvalov et al. and Kodama et al. included retrospective studies; similarly Larsson et al. focused on prospective studies), ii) only general population cohorts (Larsson et al. included one cohort with pre-existing CVD), iii) only incident AF events (Kodama et al. included studies on AF recurrence), iv) only estimates from Cox or Poisson regression (Samokhvalov et al., Kodama et al., and Larsson et al. all included estimates from logistic regression), v) only the most adjusted alcohol estimate per cohort (Samokhvalov et al. included the study with the most comprehensive alcohol data, while Larsson et al. did not report an approach to selecting from multiple estimates per cohort), and lastly vi) our more recent review and more inclusive field synopsis method includes eight reports that have not been included in the previous reviews (28, 35, 46, 53, 70, 79, 83, 88). Based on the three direct alcohol associations we identified, the increased risk of developing AF was confined to the highest alcohol intake levels, as opposed to there being a J–shaped or dose–response relationship. Overall, our findings indicate that at present, there is limited consistent evidence on which recommended alcohol intake levels for primary prevention of AF could be based.

### Discordant associations

We found some evidence that white ethnicity, taller height, lower total cholesterol and lower diastolic blood pressure might confer a higher risk of incident AF, which is in the opposite direction to their known associations with incident CHD (12). Our findings regarding cholesterol suggest that reducing cholesterol may not be relevant for the primary prevention of AF, and are in line with an existing meta–analysis of trial evidence, which did not support the role of statins for prevention of AF in participants with underlying CVD (102). Previously, it been demonstrated that blood pressure has markedly different associations with the incidence of 12 individual cardiovascular diseases (not including AF) (103). We now provide some, albeit mixed, evidence that this may also be the case for AF. The direct and inverse associations shown for systolic and diastolic blood pressure respectively, may indicate high pulse pressure, which is a marker of arterial stiffness and is more prevalent in older populations (104). Two earlier studies found an association between pulse pressure and incidence of AF (69, 84); however, pulse pressure was not considered in this review as its clinical utility is not well defined (105).

### Clinical implications

The observational evidence summarised here suggests that programmes for AF primary prevention may need to differ slightly from those which have guided clinicians and public health practitioners in the primary prevention of other CVDs. Existing management strategies to tackle obesity, smoking, alcohol and hypertension may have a role but the current evidence is insufficient to design AF specific interventions. The risk factors included in available prediction tools for 5- or 10-year risk of incident AF are supported by our systematic review, and these tools should be used more frequently in clinical practice (47, 70). Such risk prediction tools could identify high–risk individuals for inclusion in primary prevention trials in AF, where there is the largest knowledge gap.

### Overall characteristics of the field

Overall, we found a relatively “young” field, which has been rapidly expanding over the last five years (see Suppl. Figure S20, Appendix p 30). Although we included 32 cohorts of 20 million participants and 600,000 AF events, we found a limited number of reports (between 3 and 19) per risk factor. Although we identified some efforts at pooling studies (e. g. the CHARGE–AF consortium of 5 cohorts, 3 countries, and 1771 AF events (47)), the amount of evidence available is markedly smaller than the scale of cohort evidence available on risk factors for CHD or stroke incidence (e. g. The Emerging Risk Factor Collaboration consists of over 100 cohorts (106)). Next, we found that the AF field is beginning to span both consented population and electronic health record studies, with all seven EHR reports published in 2011–2015. In the era of “big data” research, EHRs offer the potential for studying associations at much larger scale, at population–level, in comparison with other risk factors, and across a wide range of diseases (107).

**What is known about this topic?**

- Atrial fibrillation is the world’s most common heart rhythm disorder, and leading cause of fatal and disabling strokes, yet current clinical practice guidelines offer no recommendations for primary prevention in individuals without pre-existing cardiovascular disease.
- Established primary prevention strategies of other cardiovascular diseases (e. g. coronary heart disease and stroke), are based on understanding of risk factors, but whether the same risk factors are associated with incident atrial fibrillation remains unclear.
- There is a lack of systematic reviews and field synopses of risk factors for atrial fibrillation among general populations and populations initially free from diagnosed CVD.

**What does this paper add?**

- A systematic evaluation of evidence from 28 consented and four electronic health record cohorts confirms the importance of hypertension and obesity, but suggests important differences in the risk factors for incident atrial fibrillation as compared with other cardiovascular diseases.
- Non-white ethnicity, shorter height, higher cholesterol and higher diastolic blood pressure all showed some evidence of being associated with lower risk of incident AF. This contrasts with the known associations of these risk factors in the opposite direction with coronary heart disease.
- The evidence for the widely held clinical opinion that alcohol use is associated with incident AF in the primary preventative setting was modest.
- These findings provide a systematic basis on which to direct research into the primary prevention of AF.

None of the EHR cohorts analysed continuous measures of blood pressure, lipids, BMI, or renal function. Linking data from consented population and EHR sources therefore represents an important research opportunity to investigate risk factors for AF at depth and at scale. Finally, we found considerable heterogeneity in study design and reporting, and a lack of consistent approach to adjustment for other risk factors (visualised as a “Swiss cheese”). Field synopses allow for differences in study designs, however in order to further inform primary preventive programmes and estimate the precision effect of each risk factor in meta-analyses; there is a need for large–scale strategic co–ordination of the field of AF prevention research.

### Strengths and limitations

The principle strength of our study – evaluation across a comprehensive range of risk factors – is also the principle weakness. In order to evaluate the breadth of the field there is a necessary restriction in the depth of analysis of any one risk factor, or relations between them. As we only searched the PubMed database, it is possible that we may have missed relevant studies. We conducted a

Supplementary Material (Appendix) is available online at www.thrombosis-online.com.

sensitivity analysis for the year 2013, and found no further eligible studies in EMBASE, which is consistent with other reports showing limited additional value of searching biomedical databases beyond PubMed (108, 109). There are of course other publications in support of searching multiple databases to identify further studies (110, 111). However, as we did not perform meta-analysis, we have not introduced any computational bias in to the present work and therefore consider our results and conclusions unlikely to change. Field synopses provide a systematic foundation, unbiased by a particular interest in one or more risk factors (112), for hypothesis generation and further research. One example of this would be to evaluate the extent to which the findings in relation to ethnicity, height and lipids (113) might be inter–related.

### Conclusions

A systematic evaluation of the available evidence suggests similarities as well as important differences in the risk factors for AF as compared with other cardiovascular diseases. This has implications for the primary prevention of AF.

## Figures and Tables

**Figure 1:**
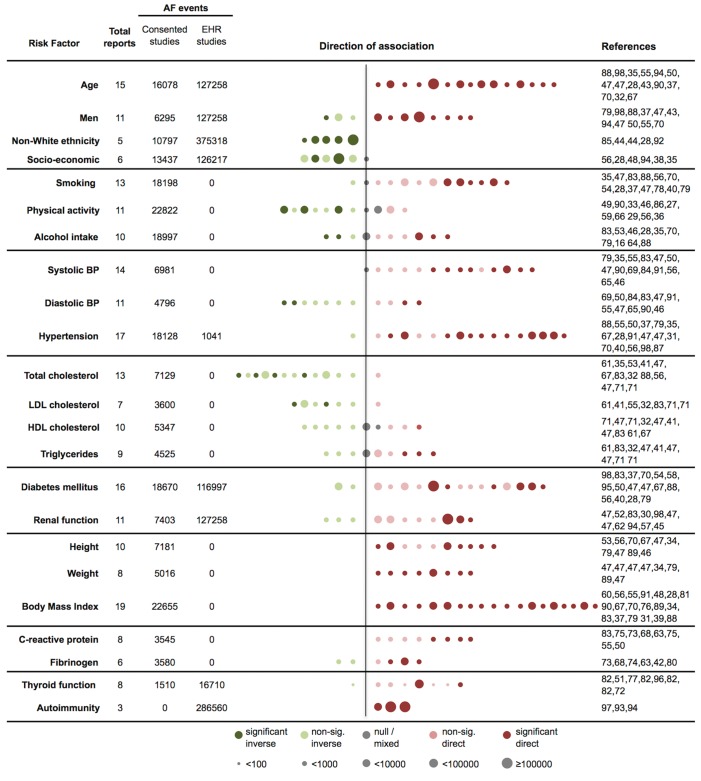
**Associations of 23 risk factors and incidence of atrial fibrillation according to number of reports, number of events, and direction of association.** AF – atrial fibrillation, BP – blood pressure, EHR – electronic health record, HDL – high-density lipoprotein, LDL – low-density lipoprotein, sig. – significant. Risk factor and reference group definitions are detailed in individual risk factors plots (Figures 2–6 and Suppl. Figures S2–S19 in Appendix, available online at www.thrombosis-online.com). Each dot represents one report, colour–coded to indicate the direction of association, and in order of most extreme inverse to most extreme direct point estimate. Dots are scaled by the number of AF events (<100, 100–<1000, 1000–<10000, 10000–<100000, or ≥100000). References correspond to each dot from left to right sequence. Associations are classified as inverse (relative risk (RR) <1.00), null or mixed (RR=1.00 or show opposite associations among subpopulations), or direct (RR>1.00). Association were regarded as significant if the 95 % CI did not cross unity.

**Table 1:** 23 cardiovascular risk factors investigated for their associations with incident atrial fibrillation in populations based cohorts.

Demographic factors
Age
Sex
Ethnicity
Socio–economic status
Health behaviours
Smoking
Physical activity
Alcohol intake
Blood pressure
Systolic blood pressure
Diastolic blood pressure
Hypertension
Cholesterol
Total cholesterol
Low–density lipoprotein cholesterol
High–density lipoprotein cholesterol
Triglycerides
Metabolic
Diabetes mellitus
Renal function
Anthropometry
Height
Weight
Body Mass Index
Inflammation
C–reactive protein
Fibrinogen
Thyroid function
Autoimmune diseases
